# Multiple Species of *Trichosporon* Produce Biofilms Highly Resistant to Triazoles and Amphotericin B

**DOI:** 10.1371/journal.pone.0109553

**Published:** 2014-10-31

**Authors:** Isabel Antonieta Iturrieta-González, Ana Carolina Barbosa Padovan, Fernando César Bizerra, Rosane Christine Hahn, Arnaldo Lopes Colombo

**Affiliations:** 1 Laboratório Especial de Micologia, Disciplina de Infectologia, Universidade Federal de São Paulo, São Paulo, SP, Brazil; 2 Departamento de Microbiologia e Imunologia, Instituto de Ciências Biomédicas, Universidade Federal de Alfenas, Alfenas, MG, Brazil; 3 Laboratório de Micologia, Faculdade de Medicina, Universidade Federal do Mato Grosso, Cuiabá, MT, Brazil; University of Wisconsin Medical School, United States of America

## Abstract

Invasive infections caused by *Trichosporon* spp. have increased considerably in recent years, especially in neutropenic and critically ill patients using catheters and antibiotics. The genus presents limited sensitivity to different antifungal agents, but triazoles are the first choice for treatment. Here, we investigated the biofilm production and antifungal susceptibility to triazoles and amphotericin B of 54 *Trichosporon* spp. isolates obtained from blood samples (19), urine (20) and superficial mycosis (15). All isolates and 7 reference strains were identified by sequence analysis and phylogenetic inferences of the IGS1 region of the rDNA. Biofilms were grown on 96-well plates and quantitation was performed using crystal violet staining, complemented with Scanning Electron Microscopy (SEM). Susceptibility tests for fluconazole, itraconazole, voriconazole and amphotericin B were processed using the microdilution broth method (CLSI) for planktonic cells and XTT reduction assay for biofilm-forming cells. Our results showed that *T. asahii* was the most frequent species identified (66.7%), followed by *T. faecale* (11.1%), *T. asteroides* (9.3%), *T. inkin* (7.4%), *T. dermatis* (3.7%) and one *T. coremiiforme* (1.8%). We identified 4 genotypes within *T. asahii* isolates (G1, G3, G4 and G5) and 2 genotypes within *T. faecale* (G1 and G3). All species exhibited high adhesion and biofilm formation capabilities, mainly *T. inkin*, *T. asteroides* and *T. faecale.* Microscopy images of high biofilm-producing isolates showed that *T. asahii* presented mainly hyphae and arthroconidia, whereas *T. asteroides* exhibited mainly short arthroconidia and few filaments. Voriconazole exhibited the best *in vitro* activity against all species tested. Biofilm-forming cells of isolates and reference strains were highly resistant to all antifungals tested. We concluded that levels of biofilm formation by *Trichosporon* spp. were similar or even greater than those described for the *Candida* genus. Biofilm-forming cells were at least 1,000 times more resistant to antifungals than planktonic cells, especially to voriconazole.

## Introduction

*Trichosporon* spp. are basidiomycetous yeast-like organisms that are widely distributed in nature, and can generally be isolated from soil, water, decomposing matter, and bird and bat droppings [1]. Occasionally, it can be part of the transient human microbiota, mainly in the skin, nails and mucosa of the respiratory and gastrointestinal tracts [1], [2]. According to data provided by the ARTEMIS DISK collection, *Trichosporon* spp. are considered to be the second or third most commonly isolated yeast species in clinical laboratories, representing 5.5 to 10.6% of all isolates [3], [4], [5].

*Trichosporon* sp. has been classically associated with white *piedra* and hypersensitivity pneumonitis syndrome [1], [6], [7], [8], [9]. However, in recent decades, *Trichosporon* sp. has been recognized as an important pathogen of systemic infections affecting immunocompromised patients, mainly those with hematological malignancies and neutropenia, as well as critically ill patients with a central venous catheter in place [10], [11], [12], [13].

The genus contains 51 species, 16 of which are able to infect human hosts [1]. Species identification based on phenotypic methods usually generates inconsistent results, and none of the commercially available tests includes new species in the genus in their databases [14]. Consequently, sequencing of the Intergenic Spacer 1 (IGS1) of the rDNA, which presents high variability within the genus, is a robust method to identify medically important *Trichosporon* species [15], [16].

There are only a few studies addressing virulence factors in *Trichosporon* spp., including biofilm production [17], [18], [19]. In addition, there is a lack of information on the antifungal susceptibility of *T. asahii* and clinically relevant emergent species within the genus. Most studies have adapted the Clinical and Laboratory Standards Institute (CLSI) microdilution method standardized for testing *Candida* spp. and *Cryptococcus* spp. to evaluate the *in vitro* antifungal susceptibility of *Trichosporon* spp. [12], [20], [21], [22]. Available data suggest that triazoles appear to be the most effective antifungal class against *Trichosporon* spp. [21], [23], [24], [25].

Different species within a genus may have biological peculiarities regarding their virulence mechanisms and drug susceptibility. In this study, we evaluated the biofilm formation and antifungal susceptibility profile of 54 clinical isolates of *Trichosporon* spp., whose species identification was confirmed by sequencing the IGS1 region of the rDNA.

## Materials and Methods

### Isolates and growth conditions

We tested a total of 54 clinical isolates of *Trichosporon* spp. obtained from different patients assisted between 2001 and 2010. Due to the retrospective character of the study, no informed consent was required for the IRB Commitee approval. Ethics Committee number 1497/11. The strains were obtained from blood (19), urine (20) and superficial sites (skin and hair) (15). Isolates were sent to the Laboratório Especial de Micologia, Universidade Federal de São Paulo, São Paulo, Brazil, for identification and antifungal susceptibility testing. In addition, the following 7 *Trichosporon* spp. and 2 *Candida* spp. reference strains were added as controls: *T. asahii* (CBS 2479 e CBS 7631), *T. inkin* (CBS 5585), *T. dermatis* (CBS 2043), *T. faecale* (CBS 4828), *T. mucoides* (CBS 7625), *T. ovoides* (CBS 7556), *C. parapsilosis* (ATCC 22019) and *C. krusei* (ATCC 6258). All isolates were stored at −80°C in liquid YEPD medium plus 20% glycerol. Prior to all experiments, the isolates were transferred twice in CHROMagar *Candida* for 48 h at 35°C to evaluate their viability and purity.

### Screening of the *Trichosporon* genus

Phenotypic identification of the genus relied on the macro and micromorphological (presence of arthroconidia) characteristics of the colonies as well as a positive urea hydrolysis test [26].

### Molecular identification of *Trichosporon* sp. isolates

Molecular identification was performed by amplification and sequencing of the IGS1 region from the rDNA according to the protocol previously described [27]. Sequencing of 2 to 4 reads per isolate was performed with the BigDye T'erminator Kit (Applied Biosystems) protocol, and the final sequence was obtained after alignment and edition in SequencherTM 4.1.4 - Gene Codes. Final species identification was obtained by comparisons with the NCBI database (http://ncbi.nlm.nih.gov) using the BLASTn tool. We considered as criteria for species identification identity and sequence coverage ≥ 98% and E-value <10^−5^ [27], [28].

### Genotyping analysis

Sequences of the IGS1 region of rDNA from all identified *T. asahii* and *T. faecale* isolates were aligned with sequences of species deposited in the GenBank (http://www.ncbi.nlm.nih.gov/genbank/) to check for the presence of genotypes 1 to 12 in *T. asahii* and 1 to 3 in *T. faecale*. Alignments were performed using the muscle algorithm implemented by SEAVIEW 4.2.12 and adjusted by eye before haplotype and phylogenetic analyses [29]. Haplotype analysis was implemented in the DNAsp 5.0, excluding gap positions [30]. Median-joining network was constructed and visualized using the software Network 4.610 [31]. Bayesian analysis was performed in MrBayes 3.02 [32].

### Biofilm formation

To assess biofilm formation of *Trichosporon* spp. isolates in a 96-well plate format, we standardized a protocol adapted from Jin et al. (2003) and di Bonaventura et al. (2006) [33], [34]. We tested different protocol conditions, such as: inoculum size (10^5^, 10^6^ and 10^7^ cells/ml), adhesion time (60, 90 and 120 min), and biofilm maturation (48 and 72 h) with static or shaking (75 rpm) incubation at 37°C. We used the isolates *C. tropicalis* LEMI 651 and *C. metapsilosis* LEMI 1799 as control strains because they were previously characterized as being high and low biofilm producers, respectively, by Melo et al. (2011) [35]. Clinical isolates and control strains were cultured in YPD at 35°C for 48 h and further subcultured under agitation in RPMI 1640 medium (pH 7.0 - MOPS) overnight at 37°C. Cells were collected by centrifugation and washed twice with sterile PBS. Cells were resuspended in RPMI 1640 (pH 7.0 - MOPS) and the inoculum adjusted to 0.4 O.D. at 530 nm (A_530_), which corresponds to 10^7^ cells/ml. Inocula of 100 µl of the cell suspensions were added to 96-well polystyrene plates with flat bottoms (Techno Plastic Products, TPP, Switzerland), using 8 wells per isolate. Plates were incubated at 37°C for 90 min with agitation at 75 rpm. After incubation, cells were aspirated, and wells were washed twice with 150 µl PBS to remove the non-adherent cells. Finally, 150 µl RPMI 1640 medium (pH 7.0 - MOPS) was added to each well, and the plate was incubated at 37°C and 75 rpm for 48 h to allow biofilm formation. Culture media were changed every 24 hours. Each experiment was performed in triplicate, and biofilm quantifications are expressed as means of 24 readings (wells) per isolate ± standard deviation.

### Biofilm quantification with crystal violet staining

Mature biofilms had the culture medium aspirated, and they were washed twice with 200 µl sterile PBS to remove non-biofilm-forming cells. For quantification using crystal violet staining, plates were dried for 45 min at room temperature. Biofilms were stained with 110 µl 0.4% aqueous crystal violet (CV) solution for 45 min. Afterwards, the CV solution was removed, and the wells were washed 3 to 5 times with 200 µL sterile distilled water. To destain the biofilms, 200 µl 95% ethanol was added, and the plate was incubated for 45 min. One hundred μl of the solution were transferred to another microplate, and the absorbance was read using a Microplate Reader 680 (BIO RAD, Hercules, USA) at 570 nm.

### Scanning electron microscopy (SEM)

Scanning electron microscopy was performed with 3 *T. asahii* and 1 *T. asteroides* isolates selected based on their biofilm production capabilities. Biofilms were formed on sterile polyvinyl chloride (PVC) strips (surface area, 0.5 cm^2^), incubated into 24-well polystyrene plates with flat bottoms (Techno Plastic Products, TPP, Switzerland), according to the protocol described previously [36]. Briefly, all biofilms formed on PVC strips were fixed overnight at 4°C with a solution composed by 4% formaldehyde and 2% glutaraldehyde buffered with 0.1 M sodium cacodylate at pH 7.2. Subsenquently, biofilms were washed repeatedly with 1% osmium tetroxide buffered with cacodylate for 1 h, then treated with 1% tannic acid for 45 min, washed three times with distilled water for 15 min, and finally, treated with 1% osmium tetroxide buffered with cacodylate for 1 h. Biofilms were dehydrated with a graded series of ethanol washes (critical-point dried in CO_2_) and coated with gold to be analyzed in the scanning electron microscope JEOL JSM-5300 (Peabody, MA, USA).

### Antifungal susceptibility testing against planktonic and biofilm-forming cells

We adapted the assay conditions of the Clinical and Laboratory Standards Institute broth microdilution method (CLSI, M27-A3/S4) to evaluate the response of planktonic fungal cells to the following drugs: fluconazole (FLC), itraconazole (ITC), voriconazole (VRC) and amphotericin B (AMB). Antifungal compounds were obtained as pure powders from the manufacturers Pfizer Inc., NY, USA and Sigma Chemical Corporation, St. Louis, MO, USA. *Candida parapsilosis* ATCC 22019 and *C. krusei* ATCC 6258 were used as controls.

Susceptibility tests for biofilm-forming cells were processed following the protocol previously described by Melo et al. (2011) [35]. Biofilms were grown for 24 h before replacement of the medium with fresh RPMI 1640 (pH 7.0 - MOPS) supplemented with the antifungals at the following concentrations: FLC (64-1024 µg/ml), ITC (1–16 µg/ml), VRC (4–64 µg/ml) and AMB (2–32 µg/ml). Biofilms were grown for 48 h and quantified afterwards using the XTT reduction assay. For the XTT (2,3-Bis-(2-Methoxy-4-Nitro-5-Sulfophenyl)-2*H*-Tetrazolium-5-Carboxanilide) reduction assay, a solution containing 200 µl PBS with 12 µl 5:1 1 mg/ml XTT (Sigma Aldrich): Menadione 0.4 mM (Sigma Aldrich) was used. The plate was incubated for 2 h at 35°C in the dark to allow XTT metabolization. Thereafter 100 µl of this solution was transferred to another microplate, and the absorbance was read using a Microplate Reader 680 (BIO RAD, Hercules, USA) at 490 nm.

Minimal Inhibitory Concentrations (MIC) of the drugs on planktonic cells were determined by visual readings after 48 h of incubation based on the lower concentration capable of inhibiting 50% of cell growth for azoles and total growth inhibition for AMB. Biofilm MICs at 48 h were defined as the lowest concentration capable of inhibiting 50% and 80% of cell growth for azoles and AMB, respectively [35].

### Statistical analysis

We performed comparisons between MIC values generated by *T. asahii* and non-*T. asahii* isolates as well as the biofilm production among different genotypes of *T. asahii* and *T. faecale*, applying the Mann-Whitney test implemented in the GraphPad Prism 6.0 software (http://www.graphpad.com/guides/prism/6/statistics/).

## Results

### Phenotypic and molecular identification of *Trichosporon* isolates

All 54 clinical and 7 reference strains of *Trichosporon* spp. included in the analysis were considered pure and viable and presented morphological and biochemical characteristics compatible with the *Trichosporon* genus. DNA sequences of the IGS1 region of the rDNA from all isolates ranged from 435 to 630 bp after contig assembly and editing. For species identification, alignments performed with the BLASTn tool successfully identified 58 of 61 *Trichosporon* spp. isolates with query coverage ≥99%, identity ≥99%, and E-value  =  0.0. Three *T. faecale* isolates (ST004B, EB087B and EB108A) generated lower identity or coverage values in the BLASTn searches, 96% and 97%, respectively. Therefore, their species identification was further confirmed by phylogenetic analysis (Figure S1). The 54 clinical isolates were identified as follows: 36 *T. asahii*, 6 *T. faecale*, 5 *T. asteroides*, 4 *T. inkin*, 2 *T. dermatis* and 1 *T. coremiiforme* (Table 1 and Table S1).

**Table 1:** Species distribution of 54 *Trichosporon* spp. clinical isolates according to the site of infection or colonization identifiyed by IGS1 rDNA sequencing.

Species	Number of Isolates/Source			Total/Percentage
	Blood	Urine	Superficial Mycosis or Skin Colonization	
*T. asahii*	12	20	4	**36/66.7%**
*T. faecale*	-	-	6	**6/11.1%**
*T. asteroides*	5	-	-	**5/9.3%**
*T. inkin*	-	-	4	**4/7.4%**
*T. dermatis*	1	-	1	**2/3.7%**
*T. coremiiforme*	1	-	-	**1/1.8%**
**Total**	**19**	**20**	**15**	**54/100%**

### Genotyping of *T. asahii* and *T. faecale* isolates

**Figure 1:**
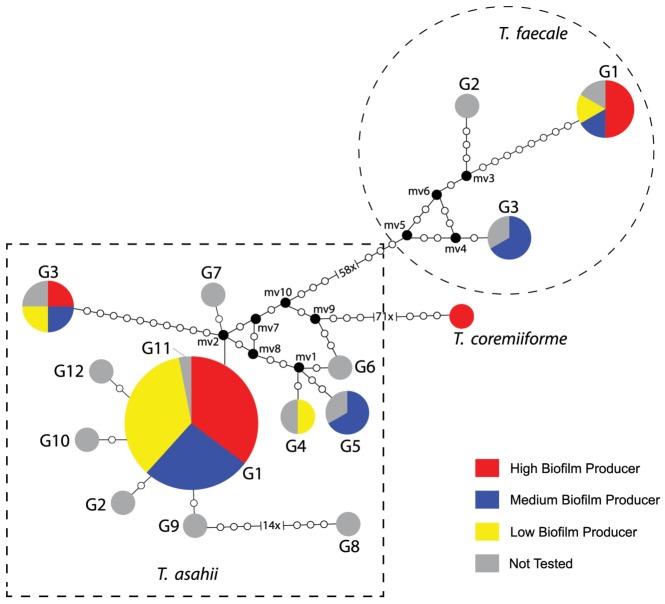
Median-joining genotypes network of *T. asahii, T. faecale* and *T. coremiiforme* based on IGS1 rDNA sequences related to biofilm quantitation. Dashed square groups the 12 different genotypes (G1 to G12) of *T. asahii*. Dashed circle groups the 3 different genotypes of *T. faecale*. Circumference sizes are proportional to the genotype frequencies. Black dots (mv = median vectors) are hypothetical missing intermediates.

Clinical isolates identified by IGS1 rDNA sequencing as *T. asahii* (36) and *T. faecale* (6) were subjected to phylogenetic analysis to confirm their identification and characterize their genotypes (Figure 1 and Figure S1). The network analysis (Figure 1) showed that 36 clinical isolates identified as *T. asahii* grouped into 4 different genotypes: 1, 3, 4 and 5. *Trichosporon asahii* G1 comprised 30 clinical isolates, the 2 reference strains CBS 2479 and CBS 7631, the G1 reference sequence (GenBank number: AB066386) and the supposedly G11 reference sequence (GenBank number: EU441160); *T. asahii* G3 consisted of 3 clinical isolates and the G3 reference sequence (GenBank number: AB066397); *T. asahii* G4 grouped 1 clinical isolate and the G4 reference sequence (GenBank number: AB180191); and *T. asahii* G5 included 2 clinical isolates and the G5 reference sequence (GenBank number: AB071387). The other reference sequences obtained from GenBank that did not grouped with our clinical isolates were: G2: AB072606, G6: AB180192, G7: AB180194, G8: AB439002, G9: AB439003, G10: EU441158, and G12: JF412789. Four of 6 *T. faecale* clinical isolates and the *T. faecale* CBS 4828 were classified as genotype 1 (G1 reference sequence number: AB066413). The isolates EB087B and EB108A had their molecular identification confirmed as *T. faecale* genotype 3 (G3 reference sequence number: AB439006). *Trichosporon faecale* G2 reference sequence (GenBank number: AB439004) did not group with any of the clinical isolates.

The consensus tree inferred by the Bayesian method (Figure S1) corroborated the network analysis, confirming the identification of *T. asahii* isolates within 4 different genotypes, including the first-time identification of the genotype 5 among Brazilian isolates and the identification of ST004B, EB087B and EB108A as *T. faecale* isolates.

### Biofilm production by *Trichosporon* spp

In a pilot study it was tested the protocol conditions for *Trichosporon* spp. biofilm formation into 96-well plates. We observed that 10^7^ cells/ml, 90 min adhesion time and 48 h biofilm growth at 75 rpm provided the most reproducible results than the other combinations (data not shown). Comparative analysis of the quantification by CV method of biofilms grown during 48 and 72 h showed that the last time point returned lower absorbance values than the first one due to biofilm detachment from the wells, mostly from isolates that were further classified as high biofilm producers (data not shown).

**Figure 2:**
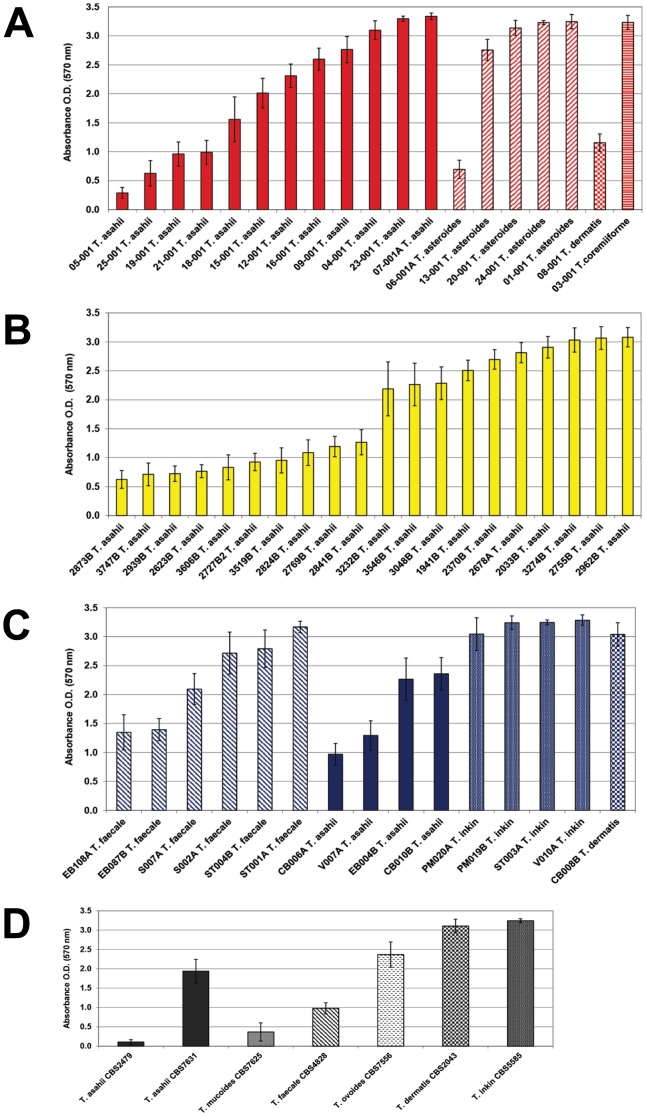
Inter and intra species variation in the biofilm production of 54 *Trichosporon* spp. clinical isolates and 7 reference strains. **A-** 19 isolates from blood identified as *T. asahii, T. asteroides, T. coremiiforme* and *T. dermatis*. **B-** 20 isolates from urine identified as *T. asahii*. **C-** 15 isolates from superficial mycosis/skin colonization identified as *T. asahii, T. dermatis, T. faecale* and *T. inkin*. **D-** 7 reference strains from CBS obtained from different sources.

The total biofilm mass of 54 clinical isolates and 7 reference strains of *Trichosporon* spp. was then assessed by CV staining after 48 h growth. Absorbance values ranged from 0.109 to 3.337. To correlate the capability for biofilm production with the different species tested and isolation sites, we arbitrarily established three categories (33.3 percentiles) of biofilm production based on the total range of the CV quantifications exhibited by the 61 isolates, categorizing the producers as follows: low: A_570_<1, medium: A_570_≥1.01 to 2.499 or high: A_570_≥2.5. Figure 2 illustrates the biofilm production by all strains tested according to the isolation source and species distribution. *Trichosporon* sp. strains were scaled from the highest to the lowest biofilm producer as *T. inkin*> *T. asteroides ≈ T. faecale*> *T. asahii.* The two *T. dermatis* isolates (classified as medium and high biofilm producers) and the single *T. coremiiforme* isolate (high biofilm producer) were not included in this rank scale due to the low number of isolates tested. When considering the source of isolation, 93.3% of the *Trichosporon* spp. strains isolated from skin were categorized as high or medium biofilm producers. Otherwise, 65% to 73.7% of the strains isolated from blood and urine, respectively, were considered high or medium biofilm producers. The reference strains were characterized as low (*T. asahii* CBS2479, *T. mucoides* CBS7625 and *T. faecale* CBS4828), medium (*T. asahii* CBS7631 and *T. ovoides* CBS7556) or high (*T. dermatis* CBS2043 and *T. inkin* CBS5585) biofilm producers.

There were no significant differences in terms of biofilm production across different genotypes identified within *T. asahii* and *T. faecale* isolates (Figure 1).

### Biofilm Scanning Electron Microscopy Analysis (SEM) of *Trichosporon* spp. isolates

**Figure 3:**
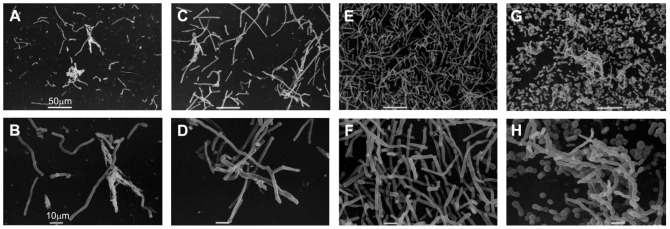
Scanning electron microscopy of 4 *Trichosporon* spp. strains grown on catheter surfaces. **A** and **B**: Low biofilm producer *T. asahii* 05-001 (CV-A_570_  =  0.287); **C** and **D**: Medium biofilm producer *T. asahii* 18-001 (CV-A_570_  =  1.557); **E** and **F**: High biofilm producer *T. asahii* 07-001A (CV-A_570_  =  3.337); and **G** and **H**: High biofilm producer *T. asteroides* 13-001 (CV-A_570_  =  2.755).

To check the efficiency of the CV method to quantify biofilms of *Trichosporon* spp., we generated SEM images of 4 *Trichosporon* spp. strains categorized as low, medium and high biofilm producers. The following isolates were analyzed: *T. asahii* 05-001 (CV-A_570_  =  0.287), *T. asahii* 18-001 (CV-A_570_  =  1.557), *T. asahii* 07-001A (CV-A_570_  =  3.337) and *T. asteroides* 13-001 (CV-A_570_  =  2.755). We observed that all *T. asahii* isolates produced micromorphology enriched with hyphae and arthroconidia regardless of the category of biofilm production (Figure 3 A to F). Notably, the *T. asteroides* strain classified as high biofilm producer exhibited a predominance of blastoconidia and arthroconidia, with a few filaments (Figure 3 G and H). We found a high correlation between SEM images and biofilm quantification by CV staining in the categorization of isolates as low or high biofilm producers.

### Susceptibility of planktonic and biofilm-forming cells of *Trichosporon* spp. isolates against 4 antifungal agents

**Figure 4:**
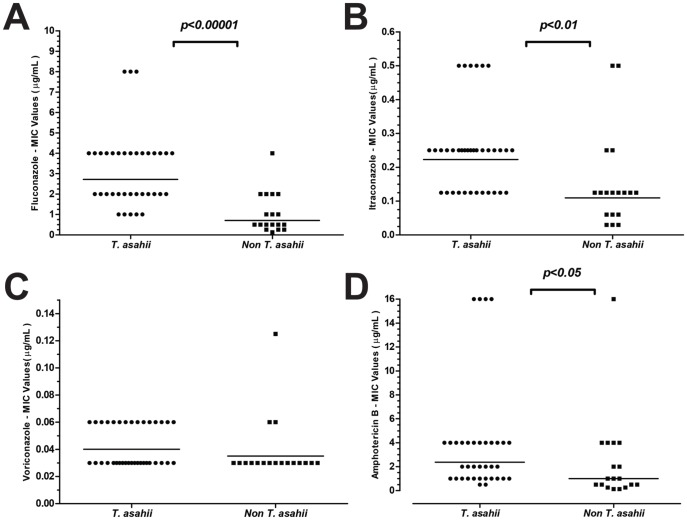
Comparative analysis of MIC values obtained against planktonic cells of *T. asahii* (36 isolates) *versus* non-*T. asahii* (18) isolates for all antifungals tested. **A-** Fluconazole; **B-** Itraconazole**; C-** Voriconazole and D- Amphotericin B.

The MIC readings of susceptibility tests for both planktonic and biofilm forming cells were performed after 48 h of incubation. Table 2 summarizes the planktonic MIC_50_, MIC_90_, and MIC ranges (μg/ml) and the geometric means (GM) obtained for the 54 clinical isolates of *Trichosporon* spp. against 4 antifungal agents. Of note, *T. asahii*, *T. asteroides* and *T. faecale* isolates exhibited MIC_50_ values for AMB ≥2 µg/ml. *Trichosporon asahii* isolates exhibited higher MIC values for all antifungals tested except for VRZ (Table 2 and Figure 4). Figure 4 depicts the comparison between MIC values obtained for *T. asahii* and non-*T. asahii* isolates. In general, significant differences were found between the two groups considering FLC, ITC and AMB, whereas VOR was the azole that exhibited the best *in vitro* activity against all *Trichosporon* species.

**Table 2:** *In vitro* activity of 4 antifungal drugs against planktonic cells of 54 clinical isolates of *Trichosporon* spp. according to the isolation site.

Species/Blood culture (Number of isolates)	MIC (μg/ml)															
	Fluconazole				Itraconazole				Voriconazole				Amphotericin B			
	Interval	MIC_50_	MIC_90_	GM	Interval	MIC_50_	MIC_90_	GM	Interval	MIC_50_	MIC_90_	GM	Interval	MIC_50_	MIC_90_	GM
*T. asahii* (12)	1–8	2	8	2.67	0.125–0.5	0.25	0.5	0.22	0.03–0.06	0.03	0.03	0.032	1–16	2	16	2.57
*T. asteroides* (5)	0.25–1	0.25	0.5	0.38	0.06–0.125	0.125	0.125	0.108	0.03	0.03	0.03	0.03	0.5–16	4	4	3.03
*T. dermatis* (1)	2	-	-	-	0.125	-	-	-	0.03	-	-	-	0.5	-	-	-
*T. coremiiforme* (1)	0.5	-	-	-	0.125	-	-	-	0.03	-	-	-	0.5	-	-	-

Species/Urine (Number of isolates)	MIC (μg/ml)															
	Fluconazole				Itraconazole				Voriconazole				Amphotericin B			
	Interval	MIC_50_	MIC_90_	GM	Interval	MIC_50_	MIC_90_	GM	Interval	MIC_50_	MIC_90_	GM	Interval	MIC_50_	MIC_90_	GM
*T. asahii* (20)	2–4	4	4	3.14	0.125–0.5	0.25	0.5	0.241	0.03–0.06	0.06	0.06	0.047	1–16	4	4	2.549

Species/Superficial Mycosis (Number of isolates)	MIC (μg/ml)															
	Fluconazole				Itraconazole				Voriconazole				Amphotericin B			
	Interval	MIC_50_	MIC_90_	GM	Interval	MIC_50_	MIC_90_	GM	Interval	MIC_50_	MIC_90_	GM	Interval	MIC_50_	MIC_90_	GM
*T. faecale* (6)	0.5–4	2	2	1.59	0.125–0.5	0.125	0.25	0.25	0.03–0.125	0.03	0.06	0.048	1–4	1	4	1.782
*T. asahii* (4)	1–2	1	2	1.41	0.125–0.25	0.125	0.125	0.149	0.03–0.06	0.03	0.03	0.036	0.5–2	0.5	1	0.841
*T. inkin* (4)	0.125–0.5	0.5	0.5	0.35	0.03–0.06	0.03	0.06	0.042	0.03	0.03	0.03	0.03	0.25	0.25	0.25	0.25
*T. dermatis* (1)	1	-	-	-	0.03	-	-	-	0.03	-	-	-	-	-	-	-

MIC_50_: Minimal inhibitory concentration capable of inhibiting growth of 50% of isolates.

MIC_90_: Minimal inhibitory concentration capable of inhibiting growth of 90% of isolates.

GM: Geometric Mean.

We were not able to compare whether the isolation site had an impact on the antifungal susceptibility profiles of the *Trichosporon* spp. strains because a clear bias of the species distribution was observed, *e.g., T. faecale* was identified only among superficial mycosis isolates, and all isolates identified in urine samples were *T. asahii*.

Table 3 summarizes MIC values for all antifungals testes against biofilm-forming cells. Regardless of the *Trichosporon* spp. species or isolation site, all strains were highly resistant to all antifungals tested.

**Table 3:** *In vitro* activity of 4 antifungal drugs against biofilm forming cells of 54 clinical isolates of *Trichosporon* spp.

Species (Number of isolates)	MIC (μg/ml)											
	Fluconazole			Itraconazole			Voriconazole			Amphotericin B		
	Interval	MIC_50_	MIC_90_	Interval	MIC_50_	MIC_90_	Interval	MIC_50_	MIC_90_	Interval	MIC_50_	MIC_90_
*T. asahii* (36)	-	>1024	>1024	-	>16	>16	-	>64	>64	-	>32	>32
*T. faecale* (6)	-	>1024	>1024	-	>16	>16	-	>64	>64	-	>32	>32
*T. asteroides* (5)	-	>1024	>1024	-	>16	>16	-	>64	>64	-	>32	>32
*T. inkin* (4)	-	>1024	>1024	-	>16	>16	-	>64	>64	-	>32	>32
*T. dermatis* (2)	-	>1024	>1024	-	>16	>16	-	>64	>64	-	>32	>32
*T. coremiiforme* (1)	-	>1024	>1024	-	>16	>16	-	>64	>64	-	>32	>32

MIC_50_: Minimal inhibitory concentration capable of inhibiting growth of 50% of isolates.

MIC_90_: Minimal inhibitory concentration capable of inhibiting growth of 90% of isolates.

## Discussion

*Trichosporon* sp. isolates are classically recognized as a cause of superficial mycoses but have recently emerged as pathogens in invasive infections, particularly in patients with acute leukemia and those subjected to invasive clinical procedures [10], [37]. It is still controversial whether there is bias in the distribution of *Trichosporon* species among different sites of infection as well as other putative biological peculiarities of the large number of recently described species within the genus, such as antifungal susceptibility and virulence. In this study, we showed that 6 *Trichosporon* species from 54 clinical isolates obtained from different types of human infections were able to form robust biofilms *in vitro* that were not susceptible to triazoles or amphotericin B.

Regarding the species distribution within different human sites, we found that *T. asahii* was the most frequently isolated species (36 of 54 isolates) in both blood and urine samples, as previously described by other studies [12], [25], [38], [39]. In addition to *T. asahii*, other publications reported that *T. mucoides* and *T. asteroides* are among the species most isolated from invasive fungal infections due to *Trichosporon* spp. [27], [40]. Curiously, we were not able to isolate any other species than *T. asahii* from urine samples. In contrast, in superficial mycosis or skin colonization, *T. inkin, T. ovoides* and *T. cutaneum* are most commonly found [1]. At this site, the majority of the isolates were identified by IGS1 rDNA sequencing as *T. faecale*, followed by *T. inkin* and *T. asahii*.

Phylogenetic analysis of IGS1 sequences confirmed the presence of 4 different genotypes among *T. asahii* isolates, with G1 being the most prevalent in Brazil as previously documented by our group [27]. Interestingly, we found isolates belonging to *T. asahii* G5 and *T. faecale* G1 and G3 for the first time among Brazilian isolates. Recently, Xia and collaborators (2012) described three new genotypes of *T. asahii:* G10, G11 and G12 [41]. In our analysis, we failure to differentiate G11 reference sequence from G1 representatives. Refinement in the analysis of genotypes including a larger number of isolates is mandatory to resolve whether or not G11 is a novel genotype or represents an artifact due to IGS1 sequencing error.

Fungal biofilm formation has gained attention among clinicians specifically due to its capability to increase mortality in patients with *Candida* spp. fungemia [42]. In our work, we quantified the *in vitro* biofilm formation of 54 clinical isolates belonging to different *Trichosporon* species by using the crystal violet staining method. We verified that isolates representative of the 6 *Trichosporon* species tested have a high production of biofilms with quantification values similar to or higher than those described for *Candida* spp. In addition, it seems that *Trichosporon* spp. are able to produce much more biofilm than other basidiomycetes, such as *Rhodotorula* spp. and *Cryptococcus* spp. [35], [36], [43], [44]. Despite some intraspecific variations in biofilm production, it is clear that more than half of the isolates representative of different *Trichosporon* species were considered to be high biofilm producers in blood, urine and skin samples. Interestingly, non-*T. asahii* isolates obtained from superficial samples produced the same amount or even more biofilms compared with strains isolated from blood samples. This finding may reflect the need for high adherence and biofilm formation to promote colonization and infection on the skin and in hair samples of human hosts. In the literature, there are only 3 published studies evaluating slime or biofilm production, and these exclusively test *T. asahii* isolates [19], [34], [45]. Therefore, to our knowledge, this is the first time that it has been shown that non-*T. asahii* isolates are high biofilm producers.

As already demonstrated with *Candida* spp. and *Rhodototula* spp. strains [35], [36], CV is a reliable tool to accurately quantify the whole biofilm mass produced by fungal cells. Indeed, we succeeded in demonstrating that the protocol for biofilm formation on 96-well plates and the quantification by CV staining exhibited good correlation with SEM images.

After adapting the CLSI protocol for antifungal susceptibility testing of planktonic cells, we found that AMB has limited *in vitro* antifungal activity against *T. asahii, T. asteroides* and *T. faecale.* Not surprisingly, we found that *T. asahii* isolates exhibited higher MICs for FLC, ITC and AMB that non-*T. asahii* isolates, corroborating the results of other studies [22], [24], [27], [46]. As already observed, VRC presented the best antifungal activity across all species tested [24], [46]. Considering that triazoles are the antifungal agents used for first-line treatment of infections caused by *Trichosporon* spp., the presence of isolates with high MICs for this drug class is disturbing. The susceptibility of biofilm-forming cells demonstrated that all 6 species were intrinsically resistant to all antifungal agents tested, independent of genotype, isolation site and biofilm quantification. Considering that voriconazole is supposed to be the best alternative to treat deep-seated infections caused by *Trichosporon* spp., it is disturbing to observe that biofilm-forming cells presented MICs at least 1,000 times higher than planktonic cells. Indeed, serum concentrations of the four antifungal agents tested are substantially lower than the MIC values that we obtained with all *Trichosporon* spp. biofilm forming cells. Recently, Sun et al. (2012) [19] provided data suggesting that triazoles, AMB and 5-FC are all inefficient against *T. asahii* biofilms.

In conclusion, we succeeded in demonstrating that not only *T. asahii* but also 5 other species of the genus are able to produce high amounts of biofilms that impair the efficacy of all antifungal drugs against biofilm-forming cells. Further studies are necessary to evaluate whether this finding may correlate with the poor clinical response observed in a substantial number of patients with deep-seated infections due to emergent *Trichosporon* spp.

## Supporting Information

Figure S1 Phylogenetic tree and genotypes of 36 *T. asahii* and 6 *T. faecale* isolates inferred by the Bayesian method. Reference sequences obtained from GenBank of *T. asahii* were G1: AB066386, G2: AB072606, G3: AB066397, G4: AB180191, G5: AB071387, G6: AB180192, G7: AB180194, G8: AB439002, G9: AB439003, G10: EU441158, G11: EU441160, and G12: JF412789. Reference sequences obtained from GenBank of *T. faecale* genotypes were: G1: AB066413, G2: AB439004, and G3: AB439006.
(EPS)

Table S1 Clinical and microbiological data of 61 *Trichosporon* sp. isolates molecularly identified by sequence analysis of the IGS1 region.
(DOC)
