# Supplementary material for: Multiple Species of Trichosporon Produce Biofilms Highly Resistant to Triazoles and Amphotericin B
Source: PLoS One. 2014 Oct 31;9(10):e109553. doi: 10.1371/journal.pone.0109553 (PMC4215839; doi:10.1371/journal.pone.0109553)
Supplement: Table S1 — Clinical and microbiological data of 61 Trichosporon sp. isolates molecularly identified by sequence analysis of the IGS1 region. (DOC) [file pone.0109553.s002.doc]

**Table S1: Clinical and microbiological data of 61 *Trichosporon* sp. isolates molecularly identified by sequence analysis of the IGS1 region.**

| **Clinical isolates and Reference strains** | **Laboratory number** | **Species identification and genotype** | **Isolation site** | **Country/State** | **Year of isolation** | **GenBank accession numbers** |
| --- | --- | --- | --- | --- | --- | --- |
| 01-001 | L01-001 | *T. asteroides* | Blood | Brazil/São Paulo | 1997/98 | KM488272 |
| 03-001 | L03-001 | *T. coremiiforme* | Blood | Brazil/São Paulo | 1997/98 | KM488273 |
| 04-001 | L733 | *T. asahii*  G1 | Blood | Brazil/São Paulo | 1998 | EU934808 |
| 05-001 | L773 | *T. asahii* G1 | Blood | Brazil/São Paulo | 1998 | EU934809 |
| 06-001A | L936 | *T. asteroides* | Blood | Brazil/São Paulo | 1998 | EU938059 |
| 07-001A | L2585 | *T. asahii* G1 | Blood | Brazil/São Paulo | 2001 | EU934810 |
| 08-001 | L08-001 | *T. dermatis* | Blood | Brazil/São Paulo | 2001 | KM488274 |
| 09-001 | L3324 | *T. asahii* G3 | Blood | Brazil/São Paulo | 2001 | EU934811 |
| 12-001 | L5826 | *T. asahii* G1 | Blood | Brazil/São Paulo | 2003 | EU938047 |
| 13-001 | L5927 | *T. asteroides* | Blood | Brazil/São Paulo | 2003 | EU938048 |
| 15-001 | L6110 | *T. asahii* G1 | Blood | Brazil/Rio Grande do Sul | 2003 | EU938049 |
| 16-001 | L16-001 | *T. asahii* G1 | Blood | Brazil/São Paulo | 2003 | KM488275 |
| 18-001 | L7730 | *T. asahii* G1 | Blood | Brazil/São Paulo | 2004 | EU938051 |
| 19-001 | L8036 | *T. asahii* G1 | Blood | Brazil/São Paulo | 2004 | EU938052 |
| 20-001 | L8064 | *T. asteroides* | Blood | Brazil/São Paulo | 2004 | EU938053 |
| 21-001 | L8722 | *T. asahii* G1 | Blood | Brazil/Brasília | 2004 | EU938054 |
| 23-001 | L9322 | *T. asahii* G1 | Blood | Brazil/São Paulo | 2005 | EU938056 |
| 24-001 | L9381 | *T. asteroides* | Blood | Brazil/São Paulo | 2005 | EU938057 |
| 25-001 | L9593 | *T. asahii* G1 | Blood | Brazil/São Paulo | 2005 | EU938058 |
| 1941B | L1941 | *T. asahii* G1 | Urine | Brazil/São Paulo | 1997 | FJ169359 |
| 2033B | L2033 | *T. asahii* G1 | Urine | Brazil/São Paulo | 1997 | FJ169360 |
| 2370B | L2370 | *T. asahii* G1 | Urine | Brazil/São Paulo | 1997 | FJ169363 |
| 2623B | L2623 | *T. asahii* G1 | Urine | Brazil/São Paulo | 1997 | FJ169364 |
| 2678A | L2678 | *T. asahii* G1 | Urine | Brazil/São Paulo | 1997 | FJ169365 |
| 2727B2 | L2727 | *T. asahii* G1 | Urine | Brazil/São Paulo | 1997 | FJ169360 |
| 2755B | L2755 | *T. asahii* G1 | Urine | Brazil/São Paulo | 1997 | FJ172161 |
| 2769B | L2769 | *T. asahii* G1 | Urine | Brazil/São Paulo | 1997 | FJ172162 |
| 2824B | L2824 | *T. asahii* G1 | Urine | Brazil/São Paulo | 1997 | FJ172163 |
| 2841B | L2841 | *T. asahii* G1 | Urine | Brazil/São Paulo | 1997 | FJ172164 |
| 2873B | L2873 | *T. asahii* G1 | Urine | Brazil/São Paulo | 1997 | FJ172165 |
| 2939B | L2939 | *T. asahii* G4 | Urine | Brazil/São Paulo | 2001 | FJ172166 |
| 2962B | L2962 | *T. asahii* G1 | Urine | Brazil/São Paulo | 2001 | FJ172167 |
| 3048B | L3048 | *T. asahii* G5 | Urine | Brazil/São Paulo | 2001 | FJ172168 |
| 3232B | L3232 | *T. asahii* G5 | Urine | Brazil/São Paulo | 2001 | FJ172173 |
| 3274B | L3274 | *T. asahii* G1 | Urine | Brazil/São Paulo | 2001 | FJ187679 |
| 3519B | L3519 | *T. asahii* G1 | Urine | Brazil/São Paulo | 2001 | FJ187682 |
| 3546B | L3546 | *T. asahii* G1 | Urine | Brazil/São Paulo | 2001 | FJ187683 |
| 3606B | L3606 | *T. asahii* G1 | Urine | Brazil/São Paulo | 2001 | FJ187684 |
| 3747B | L3747 | *T. asahii* G1 | Urine | Brazil/São Paulo | 2001 | FJ187685 |
| ST001A | L8339 | *T. faecale* G1 | Pubic hair | Brazil/Mato Grosso | 2009 | KM488276 |
| S002A | L7923 | *T. faecale* G1 | Pubic hair | Brazil/Mato Grosso | 2009 | KM488277 |
| ST004B | L8342 | *T. faecale* G1 | Penis skin | Brazil/Mato Grosso | 2010 | KM488278 |
| S007A | L7925 | *T. faecale* G1 | Buttock skin | Brazil/Mato Grosso | 2011 | KM488279 |
| EB108A | L8333 | *T. faecale* G3 | Pubic hair | Brazil/Mato Grosso | 2009 | KM488280 |
| EB087B | L8331 | *T. faecale* G3 | Pubic hair | Brazil/Mato Grosso | 2009 | KM488281 |
| V007A | L7926 | *T. asahii* G3 | Beard hair | Brazil/Mato Grosso | 2010 | KM488282 |
| CB010B | L8327 | *T. asahii* G1 | Penis skin | Brazil/Mato Grosso | 2009 | KM488283 |
| EB004B | L8329 | *T. asahii* G1 | Pubic hair | Brazil/Mato Grosso | 2008 | KM488284 |
| CB006A | L8326 | *T. asahii* G3 | Inguinal skin | Brazil/Mato Grosso | 2009 | KM488285 |
| ST003A | L8340 | *T. inkin* | Pubic hair | Brazil/Mato Grosso | 2010 | KM488286 |
| PM019B | L8336 | *T. inkin* | Pubic hair | Brazil/Mato Grosso | 2009 | KM488287 |
| PM020A | L8337 | *T. inkin* | Inguinal skin | Brazil/Mato Grosso | 2009 | KM488288 |
| V010A | L7927 | *T. inkin* | Inguinal skin | Brazil/Mato Grosso | 2010 | KM488289 |
| CB008B | L7916 | *T. dermatis* | Pubic hair | Brazil/Mato Grosso | 2009 | KM488290 |
| CBS 2479 | CBS 2479 | *T. asahii* G1 | Skin | Japan | 1997 | EU934801 |
| CBS 7631 | CBS 7631 | *T. asahii* G1 | Blood | France | 1997 | KM488291 |
| CBS 7556 | CBS 7556 | *T. ovoides* | Hair scalp | - | 1992 | EU934805 |
| CBS 2043 | CBS 2043 | *T. dermatis* | Skin | Germany | 1997 | KM488292 |
| CBS 4828 | CBS 4828 | *T. faecale* G1 | Feces | - | 1959 | KM488293 |
| CBS 5585 | CBS 5585 | *T. inkin* | Skin | - | 1967 | EU934804 |
| CBS 7625 | CBS 7625 | *T. mucoides* | Spinal fluid | Belgium | 1992 | EU934806 |

G1: Genotype 1; G3: Genotype 3; G4: Genotype 4; G5: Genotype 5

CBS: Centraalbureau voor Schimmelcultures
